# Proximal and distal muscle fatigue differentially affect movement coordination

**DOI:** 10.1371/journal.pone.0172835

**Published:** 2017-02-24

**Authors:** Jeffrey C. Cowley, Deanna H. Gates

**Affiliations:** 1 School of Kinesiology, University of Michigan, Ann Arbor, Michigan, United States of America; 2 Department of Biomedical Engineering, University of Michigan, Ann Arbor, Michigan, United States of America; Charite Universitatsmedizin Berlin, GERMANY

## Abstract

Muscle fatigue can cause people to change their movement patterns and these changes could contribute to acute or overuse injuries. However, these effects depend on which muscles are fatigued. The purpose of this study was to determine the differential effects of proximal and distal upper extremity muscle fatigue on repetitive movements. Fourteen subjects completed a repetitive ratcheting task before and after a fatigue protocol on separate days. The fatigue protocol either fatigued the proximal (shoulder flexor) or distal (finger flexor) muscles. Pre/Post changes in trunk, shoulder, elbow, and wrist kinematics were compared to determine how proximal and distal fatigue affected multi-joint movement patterns and variability. Proximal fatigue caused a significant increase (7°, p < 0.005) in trunk lean and velocity, reduced humeral elevation (11°, p < 0.005), and increased elbow flexion (4°, p < 0.01). In contrast, distal fatigue caused small but significant changes in trunk angles (2°, p < 0.05), increased velocity of wrench movement relative to the hand (17°/s, p < 0.001), and earlier wrist extension (4%, p < 0.005). Movement variability increased at proximal joints but not distal joints after both fatigue protocols (p < 0.05). Varying movements at proximal joints may help people adapt to fatigue at either proximal or distal joints. The identified differences between proximal and distal muscle fatigue adaptations could facilitate risk assessment of occupational tasks.

## Introduction

In 2014, more than 350,000 people in the U.S. missed work due to work related musculoskeletal disorders [1]. Such injuries are caused either by a single event (acute) or the accumulation of repetitive stress (overuse) in a given area. Muscle fatigue has been implicated in both acute and overuse injuries. Fatigue impairs muscle strength [2], reaction time [3], and proprioception [4]. Due to these changes, muscle fatigue may limit the ability to respond to sudden perturbations and can cause people to alter their kinematic patterns. Changes in kinematics can affect the distribution of forces on the body, leading to injuries.

Many factors influence the way that people change their movement patterns after fatigue. In particular, fatigue that is localized in a specific muscle group causes greater changes in muscle coordination [5] and movement amplitude and speed [6] compared to fatigue that is widespread over several muscles. The direct relationship between muscle fatigue, movement, and injury is difficult to discern because the conditions that lead to fatigue and the activities performed while fatigued vary across worksites. Many complex work environments require people to perform a variety of tasks in no specific order throughout a work period. These kinds of jobs (e.g. construction, retail, health services) are among those with the highest rates of work related musculoskeletal disorders [7]. In these working conditions, tasks that cause localized fatigue of one muscle group may be closely followed by different tasks. Thus, localized muscle fatigue can affect movement kinematics during various tasks.

Changes in kinematics due to muscle fatigue depend on which joints and muscles are affected [8, 9] because different joints (and therefore muscles) are used for different task-specific objectives [10, 11]. Proximal joints are generally responsible for the overall movement pattern of the arm, while distal joints primarily fine-tune movements to achieve the task goal [11, 12]. Occupational tasks may lead to fatigue of proximal muscles (e.g. overhead lifting), distal muscles (e.g. assembly tasks), or simultaneous proximal and distal fatigue (e.g. overhead assembly). The unique functions of proximal and distal joints suggest that fatigue of proximal and distal muscles will have different effects on the movement patterns people use.

During multi-joint movement tasks, proximal or distal muscle fatigue has the potential to affect all the degrees of freedom in the kinematic chain [8, 13]. During repetitive sawing, fatigue of the elbow extensors caused the range of motion of the shoulder, trunk, and wrist to increase, while the elbow range of motion decreased [14]. In a ball-throwing task, fatigue of the finger flexors and extensors caused motion of the forearm and hand to become more synchronized [15]. Generally, prior work has shown that proximal muscle fatigue causes widespread changes in joint angles and range of motion [8, 13, 14], and distal muscle fatigue causes changes in the timing of joint motion [15]. However, differences in task objectives, muscles fatigued, and types of analyses used limit the ability to compare the results of proximal and distal fatigue from different studies. Only one study examined the effects of proximal and distal fatigue separately during the same planar disc throwing task [9]. While throwing performance was not affected by either condition, fatigue of the elbow flexors resulted in larger changes in joint kinetics and kinematics, whereas wrist extensor fatigue primarily caused changes in the timing of joint motion. Although these results support the distinct differences in fatigue of proximal and distal joints, the analysis was limited to a self-paced planar, two degrees of freedom movement. Occupational tasks often include repetitive movements that involve many degrees of freedom and external timing constraints. The results of previous work may not apply to more complex multi-joint movements like those performed in working environments.

Proximal and distal muscle fatigue may also differentially affect kinematic variability. Low variability can impair the ability to respond to perturbations and may cause tissues to be stressed repeatedly [16], thus increasing the risk of soft tissue injury. In contrast, high variability can alleviate the load on tissues by distributing the stress to different areas [17] but may also increase the risk of errors or acute injury by increasing the likelihood of extreme movements [18]. Fatigue may cause variability to increase at affected joints, and decrease at unaffected joints. For example, shoulder fatigue increased kinematic variability of the shoulder and decreased variability at distal joints during reaching movements [19] and assembly tasks [13]. While this may imply that variability specifically increases at affected joints and decreases at unaffected joints, it is also possible that increasing variability at proximal joints and decreasing variability at distal joints is a generic strategy used to compensate for muscle fatigue.

The purpose of this study was to determine the effects of localized fatigue of proximal and distal upper extremity muscles on joint kinematics and kinematic variability during a repetitive, timed, multi-joint task. There are numerous occupational tasks, and these can require varying degrees of proximal and distal joint movement. In order to compare the effects of proximal and distal fatigue on movement patterns during the same task, we selected a ratcheting task that involved a similar dynamic range of motion at the shoulder and wrist joints. Subjects completed the repetitive ratcheting task in time with a metronome before and after fatigue of either the shoulder flexors (proximal) or finger flexors (distal). The proximal (shoulder and elbow) joints were responsible for the overall ratcheting movement pattern, while the distal (finger and wrist) joints were responsible for stabilizing the wrench on the bolt. We hypothesized that fatigue of the shoulder flexors would cause greater changes in multi-joint kinematic patterns compared to fatigue of the finger flexors because proximal joints control the overall position of the arm. Secondarily, we hypothesized that movement variability of the trunk, shoulder and elbow would increase after proximal fatigue and that variability of the wrist and elbow would decrease after distal fatigue.

## Materials and methods

### Ethics statement

The University of Michigan’s Institutional Review Board approved this protocol (HUM00095995). All participants gave written informed consent prior to participation.

### Participants

Fourteen (7 female) healthy, right-handed adults participated in this study. Their mean age and BMI were 27 ± 13 (range: 18–64) years and 24.4 ± 3.3 kg/m^2^, respectively. Handedness was verified using a modified version of the Edinburgh Inventory [20]. Individuals younger than 18, older than 65, or with any history of serious musculoskeletal, cardiovascular, neurological, respiratory, or visual problems were excluded.

### Experimental protocol

**Fig 1:**
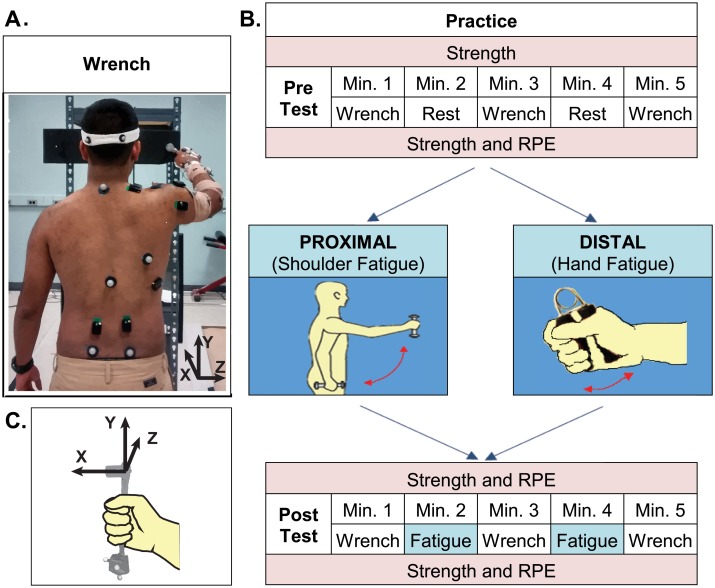
Experimental design. (**A**) Ratcheting Task. Subjects stood in front of a board placed at 60% of arm length in front of the toes, and rotated a bolt placed through the board at eye level using a ratcheting socket wrench. The torque required to rotate the bolt clockwise was ~ 4 Nm. (**B**) Experimental Session. Subjects performed three trials of a repetitive ratcheting task pre and post fatigue. Two different fatigue protocols (Proximal/Distal) were performed on separate days at least one week apart. Subjects performed the fatigue task during minutes 2 and 4 of the post-test to prevent recovery in the targeted muscle group. The order of test days was randomized. (**C**) Illustration of the wrench coordinate system.

Prior to participation, subjects answered a series of questions about their habitual activity and tool use. They then completed two experimental sessions approximately one week apart in random order. Both sessions followed the same general protocol (Fig 1). At the beginning of each session, baseline shoulder flexion and grip strength measures were recorded. Subjects then performed a pre-test consisting of three, one minute intervals of a repetitive ratcheting task (Fig 1A) alternating with one minute rest periods (Fig 1B). Following the pre-test, subjects completed one of two fatigue protocols to fatigue either proximal or distal muscles. Finally, subjects completed a post-test by performing three, one minute intervals of a repetitive work task, alternating with one minute periods in which they continued the fatiguing task. This protocol was designed to limit the development of muscle fatigue in the non-targeted muscle group during ratcheting while maintaining fatigue in the targeted muscle group. Throughout each session, muscle strength and ratings of perceived exertion (RPE) were assessed at regular intervals to measure the progression of fatigue (Fig 1B).

Subjects performed a repetitive ratcheting task before and after fatigue. The ratcheting task was designed to require similar motion amplitude at the shoulder and wrist. In this task, subjects stood in front of a board placed 60% of arm length anterior to the toes while grasping a ratcheting socket wrench (~ 0.4 kg) in the right hand (Fig 1A). Subjects repeatedly rotated a bolt placed at eye level at 1 Hz. Subjects were instructed to end each rotation in time with a metronome beat but were not given explicit instruction on how far to rotate the bolt. The primary wrench movement in this task is generated at the shoulder and elbow joints using combined humeral rotation and elevation, and forearm supination. The joints of the wrist and hand play a stabilizing role to ensure that the force generated proximally is directed properly to rotate the bolt. Specifically, the hand and wrist must maintain the appropriate position of the wrench to prevent the bolt from slipping out of the socket. To reduce learning effects, subjects practiced the ratcheting task for one minute before each data collection.

Maximum isometric shoulder flexor and grip strength were measured using hand-held dynamometers. Peak shoulder flexion force (N) was measured with subjects sitting on a stool with the right arm raised to 90 degrees of shoulder flexion. Subjects pushed upward against a hand-held load cell (Lafayette Instruments, Lafayette, IN) for 4 s. A digital output displayed the peak force in Newtons. Grip strength (kg) was measured as the peak force obtained during 4 s of maximal gripping using a hydraulic hand dynamometer (Baseline, White Plains, NY). The maximum force was displayed on a dial measurement gauge in half-kilogram increments. The average of three peak forces was taken as the participant’s maximum voluntary contraction (MVC). MVC at subsequent time intervals was expressed as a percentage of its initial value.

Static and dynamic muscle contractions may differentially affect muscle properties and fatigue [21]. Therefore, repetitive dynamic tasks were used for the proximal and distal fatigue protocols. The proximal fatigue protocol (Fig 1B) was designed to fatigue the shoulder flexors. Subjects repeatedly raised and lowered a weight (~10% max shoulder flexion strength) to shoulder height in the sagittal plane with the right arm straight at a frequency of 0.5 Hz. A custom strap was wrapped around the fingers to hold the hand closed and reduce the effort of distal muscles. The distal fatigue protocol (Fig 1B) was designed to fatigue the intrinsic and extrinsic finger and wrist muscles. The right arm was placed in a static resting position, and subjects repeatedly squeezed a spring-loaded grip trainer with their right hand at a frequency of 1 Hz. A reflective marker was used to verify that all subjects compressed the spring, but the amount of compression was not enforced. During both fatigue tasks, subjects were instructed to match their movement phase (up-down, squeeze-release) with a metronome. Fatigue was verified using ratings of perceived exertion (RPE) on the Borg CR-10 scale [22]. Subjects performed the fatigue tasks for three minutes or until they felt that they could no longer continue. If at the end of three minutes, a subject’s RPE was < 8, the subject was asked to continue the task for another three minutes or until they could not continue.

The motion of six body segments and the wrench was tracked at 120 Hz with a 16 camera motion capture system (Motion Analysis, Santa Rosa, CA) using 34 reflective markers. Pelvis motion was tracked using markers placed bilaterally on the anterior and posterior superior iliac spines. The trunk was tracked using markers on the xiphoid process, sternal notch, seventh cervical vertebra, and eighth thoracic vertebra. To track head motion, subjects wore a headband with bilateral anterior and posterior markers attached approximately in a horizontal plane. Clusters of four markers each were used to track arm and forearm motion. The hand was tracked using markers on the third and fifth metacarpal heads, diaphysis of the second metacarpal, and base of the third metacarpal. Anatomical markers were also placed on the acromion process, medial and lateral humeral epicondyles, radial and ulnar styloids, and a reference marker was placed over the right scapula. To measure the effects of fatigue on wrench movement and task execution, the position of the wrench was tracked using four reflective markers attached to the wrench (Fig 1C). Muscle activity in the right arm and trunk was recorded at 1200 Hz using 13 wireless electrodes (Delsys, Boston, MA) placed on right and left erector spinae and right side latissimus dorsi, trapezius, pectoralis major, anterior, middle, and posterior deltoid, triceps, biceps, wrist flexors, wrist extensors, and thenar muscles.

### Data analysis

A 6-segment model was created in Visual 3D (CMotion, Germantown, MA) using marker positions and joint centers, as described in [23]. Trunk-pelvis, shoulder, elbow, and wrist kinematics were calculated using Euler angles with rotation sequences recommended by the International Society of Biomechanics [24]. In this convention, three planes of shoulder movement are defined as 1) humeral plane angle which approximately corresponds to horizontal adduction/abduction, 2) humeral elevation angle, and 3) humeral internal/external rotation. In the current work, all angles are labelled according to the positive direction of movement to improve clarity. For example, negative wrist flexion, or trunk right lean angles represent wrist extension and trunk left lean, respectively. The wrench-lab angle was calculated using an X-Y-Z rotation sequence, and the wrench-hand angle was calculated using a Z-Y-X rotation sequence (Fig 1C).

**Fig 2:**
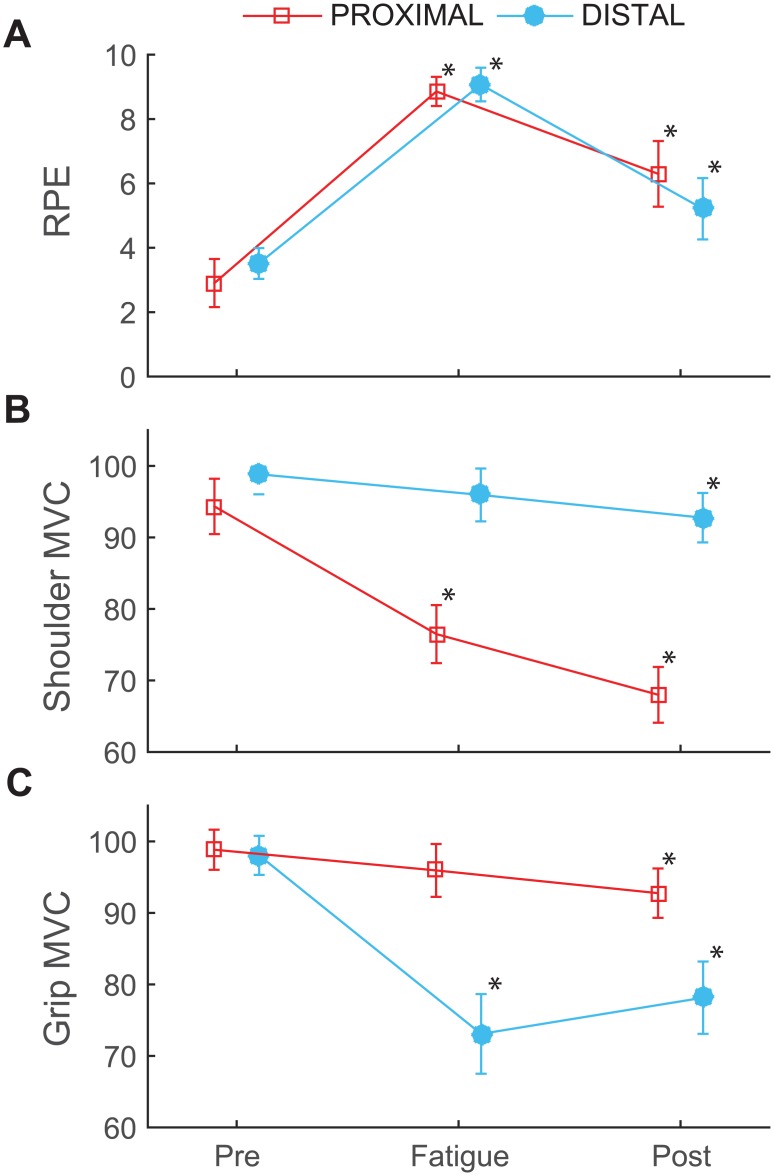
Perceived exertion and strength. (**A**) Average ratings of perceived exertion (RPE), (**B**) shoulder flexion maximum voluntary contraction (MVC), and (**C**) grip MVC for the proximal (squares) and distal (circles) fatigue sessions after the pre-test, fatigue, and post-test on each day. MVCs are reported as a percentage of the initial MVC. Error bars represent 95% confidence intervals. * indicates a difference from baseline strength.

Movement cycles were identified as the time between consecutive wrench-lab X angle minima (Fig 2A) such that the start and end of a movement occurred with the wrench at its top position. Data was time normalized to 101 points (0 to 100% of the movement cycle. We calculated the maximum and minimum angle, and the magnitude and timing (% movement cycle) of peak joint velocity for each movement. Variability of the movement pattern was calculated as MeanSD [25]: the average standard deviation of the joint angle across the movement cycle. Kinematic were averaged across all movements (3 minutes).

EMG data were bandpass filtered to a range between 20 and 450 z. The instantaneous mean power frequency (IMPF) was calculated using a continuous wavelet transform algorithm with a daubechies (‘db5’) wavelet (MATLAB 2015a, Mathworks, Natick, MA) as outlined in [26, 27]. The mean IMPF value was obtained for each movement cycle of the fatigue protocol. The rate of decrease in frequency was calculated using the average IMPF from each cycle [6]. The IMPF was expected to decrease because muscle fatigue causes motor units to contract more synchronously leading to a decrease in high frequencies in the EMG signal [27, 28].

### Statistical analysis

One sample t-tests were used to determine whether the IMPF slopes were less than zero. MVCs and RPEs were compared using 2-factor repeated measures ANOVAs to test for differences due to fatigue location (proximal vs. distal) and measurement time (pre, fatigue, post). For wrench variables (rotation, repetitions, variability, movement time, and velocity), 2-factor repeated measures ANOVAs were used to test for differences in performance due to fatigue location (proximal vs. distal) and fatigue state (pre vs. post). For each joint (trunk-pelvis, shoulder, elbow, wrist, wrench-hand), a series of 2-factor (PROX/DIST × PRE/POST) repeated measures MANOVAs was used to test for differences in joint angle, angular velocity, timing, and variability. For each significant MANOVA, univariate statistics were obtained. Mauchly’s test of sphericity was used to test the assumption of equal variance. When Mauchly’s test was significant, a Greenhouse-Geiser correction was applied to the F statistic and χ^2^ was reported. Significant interactions were further examined using estimated marginal means with a Bonferroni adjustment for multiple comparisons. Significance level was set at p < 0.05 for all comparisons.

## Results

All subjects reached an RPE ≥ 8 during the fatiguing tasks. During shoulder fatigue, two subjects stopped before 3 minutes, seven subjects, were fatigued after 3 minutes, and five subjects continued longer than 3 minutes. During the hand fatigue task, four subjects stopped before 3 minutes, seven subjects were fatigued after 3 minutes, and 3 subjects continued longer than 3 minutes. No subject continued either task longer than 6 minutes. There was a main effect of time point (F[2,12] = 118.131; p < 0.001) and a PROX/DIST × time point interaction effect for RPE (χ^2^ = 7.124, p = 0.028; F[1.382,17.96] = 6.564, p = 0.013). During the post-test RPE was higher than the pre-test and lower than fatigue (p < 0.005). However there were no significant differences between sessions at any time point (p = 0.054; Fig 2A).

During the hand fatigue task, IMPF slopes were negative for all muscles (p < 0.05) except anterior deltoid (p = 0.751). During the shoulder fatigue task, IMPF slopes were negative for all muscles (p < 0.03) except thenar muscles (p > 0.417; S1 Dataset). MVCs declined by about 20% after both proximal and distal fatigue (F[2, 12] = 98.27; p < 0.001). There was a PROX/DIST × time point interaction for MVC (χ^2^ = 8.136, p = 0.017; F[1.34, 17.422] = 7.51, p = 0.009). After the post-test, shoulder flexion MVC (68 ± 7%) was lower than grip MVC (78 ± 10%, p = 0.005). Shoulder flexion and grip MVCs did not differ at any other time point (p > 0.19; Fig 2B and 2C). In the non-targeted muscle group, MVCs declined by about 5% from baseline to the end of the session (F[2, 11] = 7.745, p = 0.008). There was no PROX/DIST × time point interaction in non-targeted muscles (F[2, 11] = 1.323, p = 0.306) (Fig 2B and 2C; S1 Table).

**Fig 3:**
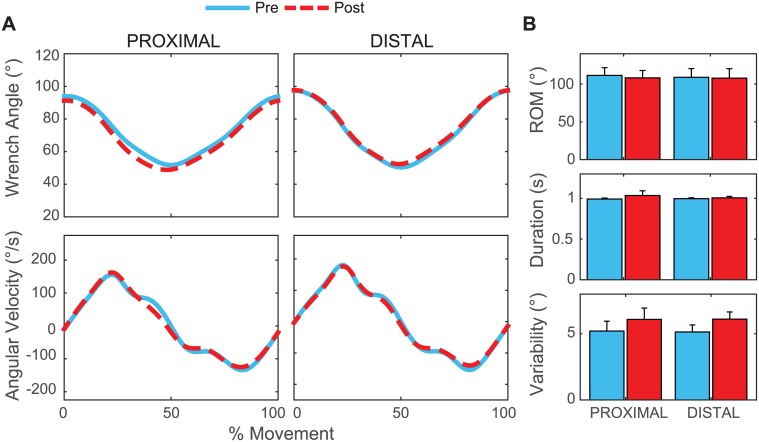
Ratcheting task execution. (**A**) Average position and angular velocity of the wrench pre (blue) and post (red) fatigue across all subjects are shown for proximal and distal fatigue. Data are normalized to 100% of the movement cycle (top position to top position). (**B**) The average range of motion (top) and movement duration (middle) of the wrench cycles did not change, but wrench rotation variability (MeanSD) increased after both fatigue protocols (bottom). Error bars show 95% confidence intervals.

There were no differences in movement time or peak speed after proximal or distal fatigue (F[1,13] < 3.659; p > 0.05) (Fig 3). The wrench angle at the top position was ~ 2 degrees lower after fatigue (F[1, 13] = 4.701, p = 0.049; Fig 3A). After fatigue, MeanSD for wrench-lab X (rotation) angle was greater (F[1, 13] = 25.611, p < 0.001), and subjects completed ~3 fewer repetitions per minute (F[1, 13] = 8.242. p = 0.013). The amplitude of wrench rotation was not affected by fatigue (F[1, 13] = 3.659, p = 0.078; Fig 3B). There were no differences between sessions and no significant PROX/DIST × PRE/POST interactions.

**Fig 4:**
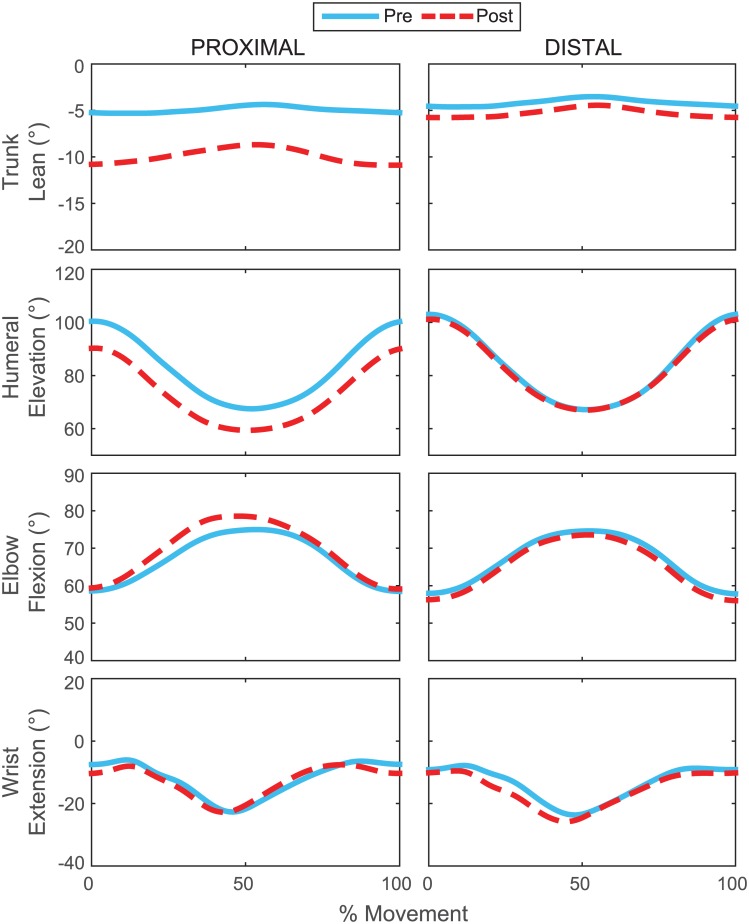
Fatigue and joint angle. Average joint angles across subjects for the ratcheting motion pre (blue) and post (red) two different fatigue protocols. Angles are normalized to 100% of the movement cycle (top position to top position). The angles shown represent those most affected by fatigue.

For peak joint angle, MANOVAs showed PROX/DIST × PRE/POST interaction effects on the trunk (F[6, 8] = 10.304), shoulder ((F[6, 8] = 11.956), and elbow joints (F[4, 10] = 9.566) (p < 0.005). Univariate analyses of these effects were significant for trunk lean, rotation and extension, humeral elevation and rotation, and elbow flexion (F[1, 13] > 4.65; p < 0.05). Humeral elevation decreased more after proximal (Pre: 101 ± 11°; Post: 90 ± 13°; p < 0.001) than distal fatigue (Pre: 103 ± 9°; Post: 102 ± 9°; p = 0.003) (Fig 4). Trunk left rotation decreased after proximal fatigue, (Pre: 5 ± 5°; Post: 4 ± 6°; p = 0.006) but increased after distal fatigue (Pre: 5 ± 4°; Post: 7 ± 4°; p = 0.037). Generally changes were smaller after proximal than distal fatigue (p < 0.02; Fig 4). Significant results from univariate analyses are summarized in Table 1.

**Table 1:** Peak angles. Maximum joint angles (degrees) are given as mean (standard deviation) across subjects.

Joint	Angle*	Proximal		Distal		P-value			
		Pre	Post	Pre	Post	Pre/Post	Prox/Dist × Pre/Post	Proximal^#^	Distal^#^
Trunk	Right Lean	6 (2)	**13 (4)**	6 (2)	**8 (2)**	< 0.001	< 0.001	< 0.001	0.003
	Right Rotation	5 (5)	**4 (6)**	5 (4)	**7 (4)**		0.017	0.006	0.037
	Extension	4 (5)	**7 (4)**	4 (5)	4 (5)	< 0.001	0.001	< 0.001	0.201
Shoulder	Humeral Plane Angle	61 (12)	60 (12)	64 (13)	63 (12)		0.057		
	Humeral Elevation	101 (11)	**90 (13)**	103 (9)	**102 (9)**	< 0.001	< 0.001	< 0.001	0.003
	Internal Rotation	-40 (10)	**-45 (9)**	-42 (9)	-41 (8)	0.031	0.004	0.001	0.506
Elbow	Pronation	-18 (18)	-14 (17)	-20 (18)	-21 (16)				
	Flexion	76 (8)	**80 (11)**	76 (9)	75 (9)	0.049	0.010	0.008	0.332
Wrist	Ulnar Deviation	30 (11)	30 (10)	23 (12)	24 (12)				
	Flexion	-26 (13)	-26 (13)	-26 (9)	-28 (10)				
Wrench-hand	X	133 (11)	134 (11)	131 (10)	130 (10)				
	Y	13 (17)	14 (16)	11 (14)	12 (13)				
	Z	79 (18)	77 (18)	73 (17)	73 (19)				

Bold values indicate a significant pre/post difference.

*Angle titles refer to the positive direction of movement.

^#^ Indicates post hoc pre/post comparison for proximal or distal fatigue only.

**Fig 5:**
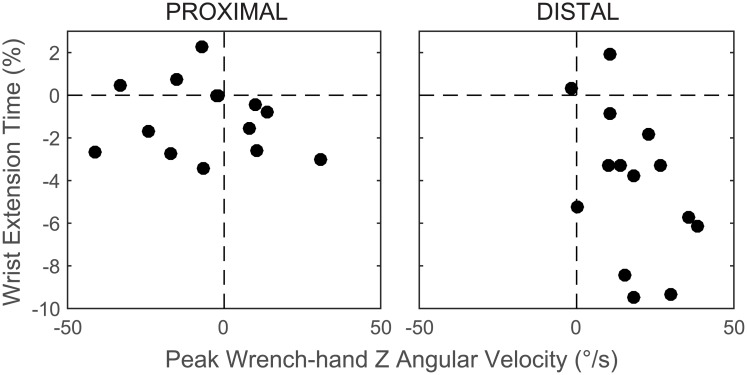
Changes in wrench-hand coordination post fatigue. The change (post—pre fatigue) in the peak angular velocity of the wrench relative to the hand about the wrench Z axis and the timing of peak wrist extension velocity for proximal and distal fatigue. Positive values indicate an increase after fatigue.

Results from MANOVAs showed main effects of fatigue on peak velocity for shoulder (F[6, 8] = 10.673, p = 0.002), elbow (F[4, 10] = 7.568, p = 0.004), and wrist (F[4, 10] = 4.626) (p = 0.023). There were also PROX/DIST × PRE/POST interaction effects on trunk (F[6, 8] = 6.159; p = 0.011) and wrench-hand velocity (F[3, 11] = 6.159; p = 0.016). There was a greater increase in trunk lean velocity after proximal (Pre: -9 ± 4°/s; Post: -15 ± 7°/s; p < 0.001) than distal fatigue (Pre: -9 ± 3°/s; Post: -11 ± 4°/s; p = 0.015). The wrench-hand Z angle had higher velocity after distal (Pre: 83 ± 34°/s; Post: 100 ± 34°/s; p < 0.001) but not proximal fatigue ((Pre: 93 ± 28°/s; Post: 88 ± 27°/s; p = 0.319) (Fig 5). Significant results are summarized in Table 2.

**Table 2:** Peak joint angular velocities. Maximum joint angular velocities (degrees/second) are given as mean (standard deviation) across subjects. Probability statistics are for univariate ANOVAs.

Joint	Angle*	Proximal		Distal		P-value			
		Pre	Post	Pre	Post	Pre/Post	Prox/Dist × Pre/Post	Proximal^#^	Distal^#^
Trunk	Right Lean	-9 (4)	**-15 (7)**	-9 (3)	**-11 (4)**	< 0.001	< 0.001	< 0.001	0.015
	Right Rotation	-15 (6)	**-22 (10)**	-15 (5)	**-17 (6)**	< 0.001	0.003	< 0.001	0.045
	Extension	-8 (3)	**-12 (6)**	-8 (3)	-10 (4)	< 0.001	0.047	0.001	0.091
Shoulder	Humeral Plane Angle	141 (49)	151 (56)	146 (60)	141 (54)				
	Humeral Elevation	126 (37)	113 (32)	140 (45)	127 (36)	0.009			
	Internal Rotation	-144 (40)	-164 (57)	-164 (41)	-164 (48)	0.068	0.095		
Elbow	Pronation	200 (67)	180 (55)	188 (56)	176 (53)	0.022			
	Flexion	93 (33)	102 (30)	94 (33)	101 (35)	0.009			
Wrist	Ulnar Deviation	146 (40)	137 (42)	139 (36)	136 (38)	0.007			
	Flexion	-143 (64)	-143 (61)	-131 (43)	-146 (48)				
Wrench-hand	X	67 (37)	68 (27)	62 (21)	66 (18)				
	Y	142 (49)	145 (46)	140 (50)	144 (59)				
	Z	93 (28)	88 (27)	83 (34)	**100 (34)**	0.09	0.001	0.319	< 0.001

Bold values indicate a significant pre/post difference.

*Angle titles refer to the positive direction of movement.

^#^ Indicates post hoc pre/post comparison for proximal or distal fatigue only.

MANOVAs revealed a significant main effect of fatigue on time of peak velocity for the elbow (F[2, 12] = 5.135, p = 0.024). Univariate analyses showed that elbow supination peak time was earlier after fatigue on both days (p = 0.01). There was a PROX/DIST × PRE/POST interaction for the wrist (F[2, 12] = 10.815, p = 0.002). Univariate analyses showed an interaction effect for wrist extension (p = 0.001). Peak wrist extension velocity occurred earlier after fatigue. This effect was larger for distal (Pre: 30.1 ± 5.5%; Post: 28.9 ± 5.7%; p = 0.001) than proximal (Pre: 32.7 ± 5%; Post: 28.6 ± 6.5%; p = 0.028) fatigue (Fig 5; S2 Table).

**Fig 6:**
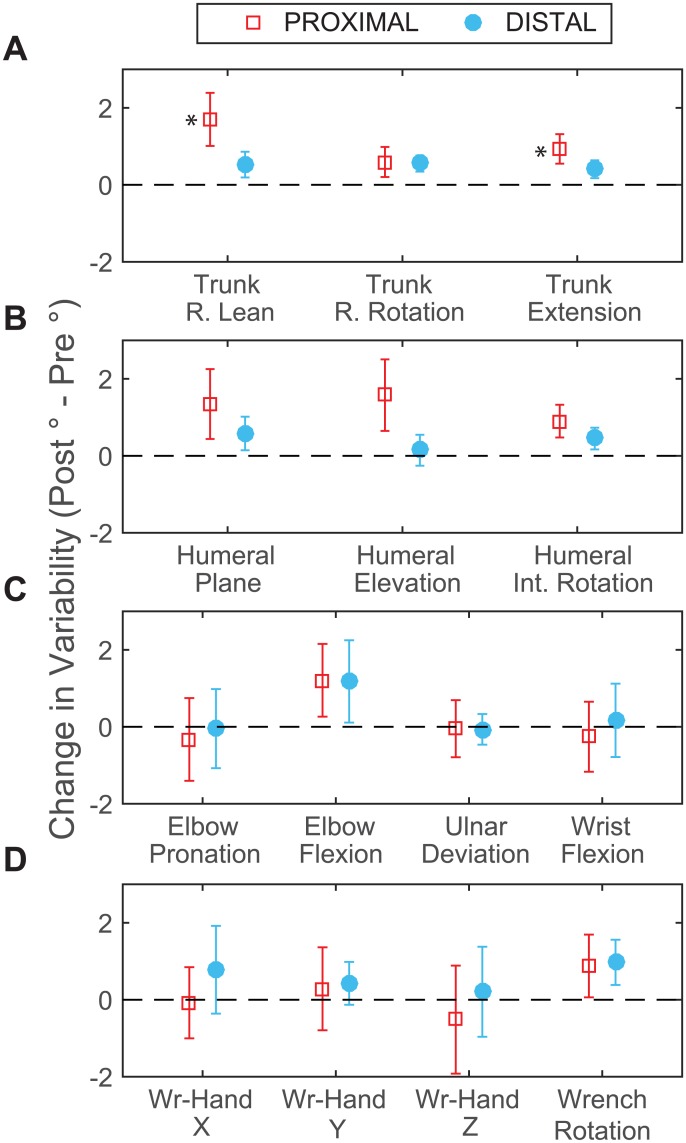
Changes in movement variability post fatigue. Change in MeanSD (post—pre fatigue) at the trunk (**A**), shoulder (**B**), elbow and wrist (**C**), and hand and wrench (**D**) after proximal (squares) and distal (circles) fatigue. Positive values indicate that variability increased after fatigue. Error bars represent the 95% confidence interval. * indicates PROX/DIST × PRE/POST interaction effect.

Results from MANOVAs showed main effects of fatigue on MeanSD for trunk (F[3,11] = 15.351, p < 0.001), shoulder (F[3, 11] = 7.481, p = 0.005), elbow (F[2, 12] = 6.077, p = 0.015), and wrench-hand angles (F[3, 11] = 4.179, p = 0.033). ANOVAs for these tests showed an increase in MeanSD after fatigue for all planes of trunk and shoulder motion (p < 0.005) and in elbow flexion (p = 0.01), but there were no significant changes for wrench-hand angles (p > 0.25). There was a PROX/DIST × PRE/POST interaction for trunk MeanSD (F[3, 11] = 4.707, p = 0.024). Univariate tests showed significant interaction effects for trunk lean (p = 0.008) and extension (p = 0.002). For these angles, MeanSD was larger after proximal (p < 0.001) than distal fatigue (p < 0.025; Fig 6; Table 3).

**Table 3:** Joint variability. MeanSD for each joint angles is given as the mean (standard deviation across subjects. Probability statistics are for univariate ANOVAs.

Joint	Angle*	Proximal		Distal		P-value			
		Pre	Post	Pre	Post	Pre/Post	Prox/Dist × Pre/Post	Proximal^#^	Distal^#^
**Trunk**	Right Lean	1.5 (0.5)	**3.2 (1.3)**	1.5 (0.3)	**1.9 (0.8)**	< 0.001	0.008	< 0.001	0.025
	Right Rotation	1.3 (0.4)	2 (0.7)	1.2 (0.3)	1.5 (0.4)	0.001	0.069		
	Extension	1.3 (0.4)	**2.6 (0.9)**	1.3 (0.4)	**1.6 (0.5)**	< 0.001	0.002	< 0.001	0.012
**Shoulder**	Humeral Plane Angle	4.6 (1.3)	6 (1.6)	4.4 (0.9)	5 (1.1)	0.004			
	Humeral Elevation	3.8 (1.4)	5.3 (1.7)	3.6 (0.8)	3.8 (0.8)	0.004			
	Humeral Internal Rotation	3.5 (0.6)	4.4 (0.9)	3.6 (0.7)	4 (0.7)	< 0.001			
**Elbow**	Pronation	5.8 (2.1)	5.5 (1.9)	6.2 (2.8)	6.2 (3)				
	Flexion	4.4 (2)	5.6 (1.1)	4.3 (1)	5.4 (1.8)	0.010			
**Wrist**	Ulnar Deviation	3.9 (1.4)	3.8 (1.2)	3.8 (1.1)	3.7 (0.8)				
	Flexion	6.3 (1.9)	6.1 (1)	6.1 (2.5)	6.3 (2)				
**Wrench-hand**	X	3.2 (1.4)	3.1 (1.1)	3.1 (1.6)	3.9 (2)	0.294			
	Y	4.7 (1.8)	5 (1.7)	4.4 (1.4)	4.8 (1.3)	0.288			
	Z	6.9 (2.6)	6.4 (2)	7.2 (3.2)	7.5 (2.5)	0.768			

Bold values indicate a significant pre/post difference.

*Angle titles refer to the positive direction of movement.

^#^ Indicates post hoc pre/post comparison for proximal or distal fatigue only.

## Discussion

This study compared the differential effects of proximal and distal muscle fatigue on movement patterns during a repetitive ratcheting task. The relative muscle strength of the non-targeted muscle group did not differ at any measurement point suggesting that the ratcheting task had a similar effect on proximal and distal muscles in the absence of a fatigue intervention. The high ratings of perceived exertion, negative IMPF slopes, and decreased maximum voluntary contractions demonstrate that subjects were fatigued after both fatigue protocols. The fatigue protocols caused MVC strength to decrease in the targeted muscle group by 18% and 24% for proximal and distal fatigue, respectively (Fig 2B and 2C), while strength in other muscle groups declined to a much smaller degree (< 4%). These results confirm that the protocols successfully fatigued the targeted muscles.

### Changes in joint kinematics

The hypothesis that fatigue of a proximal muscle group would cause greater kinematic changes than fatigue of a distal muscle group was supported. The primary effects of shoulder fatigue were reduced humeral elevation, increased elbow flexion, and increased left trunk lean angle and angular velocity. These changes are consistent with previous research and suggest a redistribution of loading to different areas [29]. Conversely, fatigue of the finger flexors caused relatively small changes in movement organization. Distal fatigue primarily affected the movement velocity and timing of the wrist and hand. The distal fatigue protocol was expected to limit the ability to stabilize the wrist, hand, and wrench. When the gripping muscles were fatigued, the wrist joint extended earlier in the movement as the subjects began to apply clockwise torque to the wrench. The early wrist extension coincided with an increase in wrench–hand velocity indicating faster movement of the wrench between the proximal interphalangeal crease of the index finger and the palmar digital crease of the thumb. The increased velocity could be caused by reduced grip force and/or changes in the relative force vector between the wrench and hand. In spite of these changes, we did not observe changes in the execution of the task or the number of movement errors.

There were significant increases in trunk-pelvis angles and angular velocity after both proximal and distal fatigue. Even small changes in trunk angles can significantly affect endpoint kinematics [30]. In particular, left rotation decreased after proximal fatigue and increased after distal fatigue. This indicates that trunk movement changed in a way that specifically compensated for the different fatigue conditions. However, changes in trunk angles were two to four times smaller after distal than proximal fatigue. Overall the results suggest that distal fatigue predominantly affects the distal joints while proximal fatigue affects all joints in the kinematic chain.

### Changes in kinematic variability

Our hypothesis that variability would increase after proximal but decrease after distal fatigue was not supported. Movement variability increased at proximal but not distal joints after both fatigue protocols. The increase in variability was larger after proximal fatigue. Results for proximal fatigue are consistent with previous studies which found increased movement variability after shoulder fatigue at proximal joints during sawing [27], reaching [19], and assembly tasks [13]. The current work expands previous findings as we found that proximal joint variability also increased when the hand muscles were fatigued. Muscle fatigue can increase neuromuscular noise and lead to increased kinematic variability. The observed increase in proximal joint variability after distal muscle fatigue might be the result of general increased descending motor drive. Alternatively, increasing kinematic variability at proximal joints might be a generic strategy to adapt to fatigue. One way to vary the load on fatigued distal muscles is to alter the pattern of proximal joint motion because this can alter the pattern of distal joint reaction torques. Thus varying proximal joint movement could change the force generated in distal muscles without necessarily varying distal joint kinematics. It is therefore possible that distal joint variability changed in ways that were not explored by the kinematic analyses used in this study (e.g. force, muscle activation patterns).

### Injury risk associated with proximal and distal fatigue

Although subjects were not explicitly told to do so, they maintained a similar movement pattern of the wrench after fatigue. After shoulder fatigue, they did this by increasing trunk movement. These changes probably served to relieve the force in the fatigued shoulder muscles, but the observed increase in trunk motion and angular velocity is likely to increase the risk of back injuries [31]. In contrast, subjects maintained similar wrench movement after hand fatigue without major kinematic changes at the joints. Prior work has shown that wrist stiffness decreases after muscle fatigue when people use power tools [32, 33]. While we did not measure stiffness here, the observed changes in wrist extension time and wrench-hand velocity suggest that stiffness decreased after distal muscle fatigue. This strategy may have helped subjects recover grip strength during the post-test. However, these changes can increase force on the hand [34], impair the ability to react to rapid forceful loading [35, 36], and may increase the risk of long term musculoskeletal disorders of the hand [33].

### Limitations

While there was no change in peak wrench velocity, the small decrease in movement repetitions may indicate that subjects moved more slowly after fatigue. However, subjects largely adhered to the instruction to follow the metronome beat. Still, there was high inter-subject variability in the way people responded to muscle fatigue. There may be large differences in fatigue for different subjects and even different muscle groups in the same subject. Some of these differences could be due to the different roles of individual muscles requiring static instead of dynamic muscle contraction. However, this is unlikely in the current study. Post hoc examination showed cyclic (dynamic) muscle activity in proximal and distal muscle groups during ratcheting.

Different work tasks could cause varying degrees of proximal and distal fatigue, but the progression and effects of the fatigue may be different for each person. Subjects may develop unique individual strategies in response to muscle fatigue. In the ratcheting task, performance stabilized within one minute of practice at the beginning of each session. However, the results demonstrate that subjects continued to modify their movement strategies throughout the sessions as they adapted to muscle fatigue. It is also likely that subjects learned and retained new movement strategies from trial to trial and day to day of the study. Some of these strategies could have helped subjects recover from fatigue. In particular, subjects began to recover from distal but not proximal fatigue during the post-test. This could be due to different movement strategies or different recovery rates of proximal and distal muscles. In addition the recovery rate might be influenced by characteristics of the fatigue task. For example, during the distal fatigue protocol, the resistance of the grip trainer was not scaled to the subject’s strength. Still, the MVCs indicate that grip strength was similarly impaired after fatigue and remained below the pre-test levels throughout the post-test indicating that the distal muscles remained fatigued.

People who use tools frequently might adapt to fatigue differently due to experience, strength, or other factors. We questioned subjects about the use of hand tools at the beginning of the experiment. One difference we observed was a change in wrist movement after distal fatigue. Wrist extension increased after hand fatigue in 10 of 14 subjects. However, in four subjects (3 male) who reported frequent tool use, wrist extension decreased. The differences in these subjects were not consistent across other measured variables, but experience with hand tools could affect both fatigue rate and movement strategy at the distal joints in particular.

Another potential factor that contributes to the fatigue response is gender. Lin et al. [37] reported that female wrists are less stiff than male wrists. In the current balanced sample of males and females, the effects of distal fatigue were larger on average in females than males. However, these differences were small and influenced by a few outliers. Even small differences between males and females may be relevant to injury risk when they occur over a large number of repetitions. Future studies should further examine the effects of experience and gender and seek to identify characteristics that cause people to adopt different movement strategies during fatiguing tasks.

## Conclusion

This study identified significant movement changes in trunk, shoulder, and elbow kinematics after proximal muscle fatigue in a repetitive, timed movement task. In contrast, after distal muscle fatigue, there were changes mainly in wrist and hand movement. Kinematic variability increased at proximal but not distal joints after both proximal and distal fatigue. These findings agree with previous research during disc throwing, and provide external validity to the idea that fatigue adaptations are governed by hierarchical control principles. Furthermore, these results underscore the importance of considering the localization of muscle fatigue in order to assess the contributions of fatigue to injury risk. Further research is needed to understand how people modify the variability of different joints during fatigue and determine how consistent these changes are across tasks.

## Supporting information

S1 Dataset Data for each individual. (XLSX)

S1 Table Maximum voluntary contraction strength. Average (SD) MVC strength (N).
(DOCX)

S2 Table Joint timing. Values are reported as percentage of movement cycle, average (SD). Probability statistics are for univariate ANOVAs.
(DOCX)
