# Supplementary material for: Proximal and distal muscle fatigue differentially affect movement coordination
Source: PLoS One. 2017 Feb 24;12(2):e0172835. doi: 10.1371/journal.pone.0172835 (PMC5325574; doi:10.1371/journal.pone.0172835)
Supplement: S1 Table — Average (SD) MVC strength (N). (DOCX) [file pone.0172835.s002.docx]

**S1 Table.** Maximum voluntary contraction (MVC) force in Newtons are given as mean (standard deviation) across the four time points.

|  | **Targeted** | | **Non-targeted** | |
| --- | --- | --- | --- | --- |
| **Time** | **Shoulder Flexion** | **Grip** | **Shoulder Flexion** | **Grip** |
| Baseline | 327 (69) | 390 (77) | 328 (65) | 379 (88) |
| Pre | 306 (58) | 382 (73) | 321 (60) | 374 (84) |
| Fatigue | 249 (55) | 281 (38) | 313 (63) | 363 (83) |
| Post | 221 (48) | 301 (47) | 309 (59) | 349 (68) |
