# Supplementary material for: Proximal and distal muscle fatigue differentially affect movement coordination
Source: PLoS One. 2017 Feb 24;12(2):e0172835. doi: 10.1371/journal.pone.0172835 (PMC5325574; doi:10.1371/journal.pone.0172835)
Supplement: S2 Table — Values are reported as percentage of movement cycle, average (SD). Probability statistics are for univariate ANOVAs. (DOCX) [file pone.0172835.s003.docx]

**S2 Table.** Time of maximum joint velocity as a percentage of movement cycle. Values are given as mean (standard deviation). Probability statistics are provided for univariate ANOVAs.

|  | | | | | | | | | |
| --- | --- | --- | --- | --- | --- | --- | --- | --- | --- |
|  |  | **Proximal** | | **Distal** | | **P-value** | | | |
| **Joint** | Angle* | Pre | Post | Pre | Post | Pre/Post | Prox/Dist × Pre/Post | Proximal^#^ | Distal^#^ |
| **Trunk** | Right Lean | 33.2 (5.5) | 30.9 (6) | 32.5 (3.6) | 33.5 (5.2) |  |  |  |  |
|  | Right Rotation | 41.2 (3.5) | 39.9 (3.2) | 41.7 (4.5) | 41 (4.2) |  |  |  |  |
|  | Extension | 26.9 (9) | 21.1 (6.3) | 26.2 (9.2) | 24.3 (8.6) |  |  |  |  |
| **Shoulder** | Humeral Plane Angle | 38 (3.5) | 36.7 (4.1) | 38.6 (3.7) | 37.1 (3.6) |  |  |  |  |
|  | Humeral Elevation | 26.1 (6.2) | 23.4 (5.2) | 25.9 (7.1) | 26.1 (7.3) |  |  |  |  |
|  | Internal Rotation | 28.4 (4.2) | 27.3 (3.9) | 28.8 (5.3) | 27.9 (4.8) |  |  |  |  |
| **Elbow** | Pronation | 28.1 (5.9) | 25.6 (5.3) | 29.2 (5) | 28.4 (6.5) | 0.010 |  |  |  |
|  | Flexion | 26.8 (6.2) | 24.9 (6.6) | 26.3 (7.2) | 26.6 (7.9) |  |  |  |  |
| **Wrist** | Ulnar Deviation | 24.6 (7.2) | 24.1 (7.9) | 25.2 (7.1) | 25.7 (7.4) |  |  |  |  |
|  | Flexion | 30.1 (5.5) | 28.9 (5.7) | 32.7 (5) | 28.6 (6.5) | 0.001 | 0.001 | 0.028 | 0.001 |
| **Wrench-hand** | X | 27.4 (5.2) | 25.7 (6.1) | 29.1 (5.3) | 28.1 (4.5) |  |  |  |  |
|  | Y | 21.9 (5.9) | 23.7 (5.8) | 22.6 (5.5) | 22.4 (6.7) |  |  |  |  |
|  | Z | 33.4 (3.9) | 32.6 (3.1) | 33.4 (4.6) | 33.1 (4.3) |  |  |  |  |

*Angle titles refer to the positive direction of movement.

# Indicates post hoc pre/post comparison for proximal or distal fatigue only.

Bold values indicate a significant pre/post difference.
